# Research and Application of Several Key Techniques in Hyperspectral Image Preprocessing

**DOI:** 10.3389/fpls.2021.627865

**Published:** 2021-02-18

**Authors:** Yu-hang Li, Xin Tan, Wei Zhang, Qing-bin Jiao, Yu-xing Xu, Hui Li, Yu-bo Zou, Lin Yang, Yuan-peng Fang

**Affiliations:** ^1^Changchun Institute of Optics, Fine Mechanics and Physics, Chinese Academy of Sciences, Changchun, China; ^2^Optical Engineering, Daheng College, University of Chinese Academy of Sciences, Beijing, China

**Keywords:** hyperspectral image, preprocessing, image segmentation, double standard reflectance plates, spatial-spectral dimension combined filtering, classification recognition

## Abstract

This paper focuses on image segmentation, image correction and spatial-spectral dimensional denoising of images in hyperspectral image preprocessing to improve the classification accuracy of hyperspectral images. Firstly, the images were filtered and segmented by using spectral angle and principal component analysis, and the segmented results are intersected and then used to mask the hyperspectral images. Hyperspectral images with a excellent segmentation result was obtained. Secondly, the standard reflectance plates with reflectance of 2 and 98% were used as *a priori* spectral information for image correction of samples with known true spectral information. The mean square error between the corrected and calibrated spectra is less than 0.0001. Comparing with the black-and-white correction method, the classification model constructed based on this method has higher classification accuracy. Finally, the convolution kernel of the one-dimensional Savitzky-Golay (SG) filter was extended into a two-dimensional convolution kernel to perform joint spatial-spectral dimensional filtering (TSG) on the hyperspectral images. The SG filter (*m* = 7,*n* = 3) and TSG filter (*m* = 3,*n* = 4) were applied to the hyperspectral image of Pavia University and the quality of the hyperspectral image was evaluated. It was found that the TSG filter retained most of the original features while the noise information of the filtered hyperspectral image was less. The hyperspectral images of sample 1–1 and sample 1–2 were processed by the image segmentation and image correction methods proposed in this paper. Then the classification models based on SG filtering and TSG filtering hyperspectral images were constructed, respectively. The results showed that the TSG filter-based model had higher classification accuracy and the classification accuracy is more than 98%.

## Introduction

In recent years, hyperspectral imaging technology has been developed increasingly mature. With the features of multiple spectral channels, high spectral resolution, strong band continuity, and “map unity,” hyperspectral imaging technology is widely used in remote sensing (Nalepa et al., 2020; Tu et al., 2020), agriculture (Jiang et al., 2016; Moliner and Romero, 2020; Zhang et al., 2020), biomedicine (Shirokanev et al., 2020; Trajanovski et al., 2020), and other fields. The data acquired by hyperspectral imaging techniques are called hyperspectral images. The analysis of hyperspectral images allows the acquisition of morphological features of external attributes and spectral features of internal component attributes of the sample, enabling the classification of the target. However, because of the high correlation of information between the bands of hyperspectral images, the following factors can interfere in practical applications.

• Redundant background information of hyperspectral images.
• Noise of dark current.
• Spatial and spectral dimensional noise.

Therefore, preprocessing operations are required before classifying hyperspectral images. Currently, methods such as thresholding (Couceiro and Ghamisi, 2016; Pang et al., 2020), region segmentation (Saravana and Rengasari, 2019) and watershed algorithms (Dao et al., 2021) are often used to remove the redundant background information from hyperspectral images. The black-and-white correction method (Esfahani et al., 2020; Xin et al., 2020) is the most widely used method for the correction of noise in dark currents. Because the hyperspectral image is a two-dimensional image in the spatial dimension, the individual bands can be denoised using classical image denoising methods; the spectral dimension is often denoised using Savitzky-Golay (SG) filtering (Wang et al., 2019; Ayaz et al., 2020; Jiang et al., 2020; Khamsopha and Teerachaichayut, 2020; Li et al., 2020; Ye et al., 2020).

Although the above preprocessing methods are widely used in hyperspectral image classification, there are still some shortcomings. When the difference between the grayscale of the region where the target of interest is located in the hyperspectral image and the grayscale between the surrounding pixels is small or the overlap of the grayscale range is large, the effect of the threshold method segmentation is often not satisfactory. Region segmentation can lead to over-segmentation or under-segmentation when the parameters are not handled properly. The essence of the watershed algorithm is the process of successive erosion of binary images, which has a high speed in image segmentation but is very prone to over-segmentation. The black-and-white correction method is divided into two steps: all-white correction and all-black correction. In the all-black correction, the fluctuation of the dark current is constantly changed by environmental factors such as temperature. Although the calibration was performed using multiple reference images and multiple dark current images to obtain the mean value (Qiu et al., 2020), the synchronization of the acquired sample spectral information with the calibration information was not guaranteed and the calibration results still had errors. Hyperspectral data are three-dimensional in nature. Although denoising in the spatial or spectral domain alone can filter out most of the noise, it is not able to handle hyperspectral images with low signal-to-noise ratio well. So joint denoising of the spatial and spectral dimensions of hyperspectral images are needed.

Firstly, to address the problem of redundant background information in hyperspectral images, this paper proposes a spectral angle joint principal component analysis (Machidon et al., 2020; Uddin et al., 2020) based on the histogram thresholding method (Datta and Chakravorty, 2018; Saravana and Rengasari, 2019) and only a few of pixels were not successfully segmented using hyperspectral images of Zhengmai 8 wheat seeds as experimental data. Secondly, in order to solve the problem of noise instability of dark current, this paper proposes the double standard reflectance plates correction method instead of the black-and-white correction method. The standard reflectance plate with 50% reflectance whose spectral information is known is used as the target sample and the standard reflectance plates with 2 and 98% reflectance are used as the calibration plates. The reflectance spectral curve after correction of the bicriteria reflectance plate is closer to the true reflectance spectral curve of the sample and the corrected result improves the total classification accuracy of the hyperspectral image by 1.88%. Finally, in order to realize the joint spatial and spectral dimension filtering of hyperspectral images, a joint spatial-spectral dimension filtering method (TSG) is proposed based on the principle of Savitzky-Golay filtering. The hyperspectral image of Pavia University was used as the experimental image to evaluate the image quality before and after SG/TSG filtering. The results show that although TSG filtering changes the original image more than SG filtering, it still retains most of the original features and has less noise information after filtering. Comparing the effects of SG/TSG filtering on the classification results, it was found that the total classification accuracy after TSG filtering was significantly greater than that after SG filtering, reaching 98.69%.

## Materials and Equipment

The variety of wheat seeds used in this experiment was Zhengmai 8, originated from Tongling, Anhui province, and provided by Taihe Experimental Station of Anhui Academy of Agricultural Sciences and Hefei Institute of Physical Sciences, Chinese Academy of Sciences. The samples were divided into two groups, one containing a number of healthy wheat seeds and the other containing a number of wheat seeds infected with scab. For healthy wheat seeds and wheat seeds infected with scab there is a breakdown in application as follows: Sample 1 was divided into two groups, Sample 1–1 and Sample 1–2. The hyperspectral images of sample 1–1 (healthy Zhengmai 8 wheat seeds) and sample 1–2 (Zhengmai 8 wheat seeds infected with scab) were used as the experimental data for image segmentation and the training set and test set for the wheat scab recognition model. The ratio of the training set to the test set is 9:1. The 4500 sample points randomly selected from the hyperspectral images of sample 1–1 and sample 1–2 are used as the training set, respectively. Then 500 sample points randomly selected from the remaining sample points are used as the test set. The same batch of wheat seeds as sample 1 but completely different from those used in sample 1 was selected as sample 2. Divide sample 2 into three groups: sample 2–1, sample 2–2, and sample 2–3. Sample 2–1 contained 240 healthy wheat seeds; sample 2–2 contained healthy wheat seeds and wheat seeds infected with scab totaling 256 seeds, 50% each; sample 2–3 contained 273 wheat seeds infected with scab. The hyperspectral images of sample 2–1, sample 2–2, and sample 2–3 are collected and used as the validation set of the classification model. The wheat of Zhengmai 8 was sown on October 10, 2016, with a fertility period of 226 days, and harvested on May 24, 2017, and the wheat was inoculated with fusarium at flowering.

**FIGURE 1:**
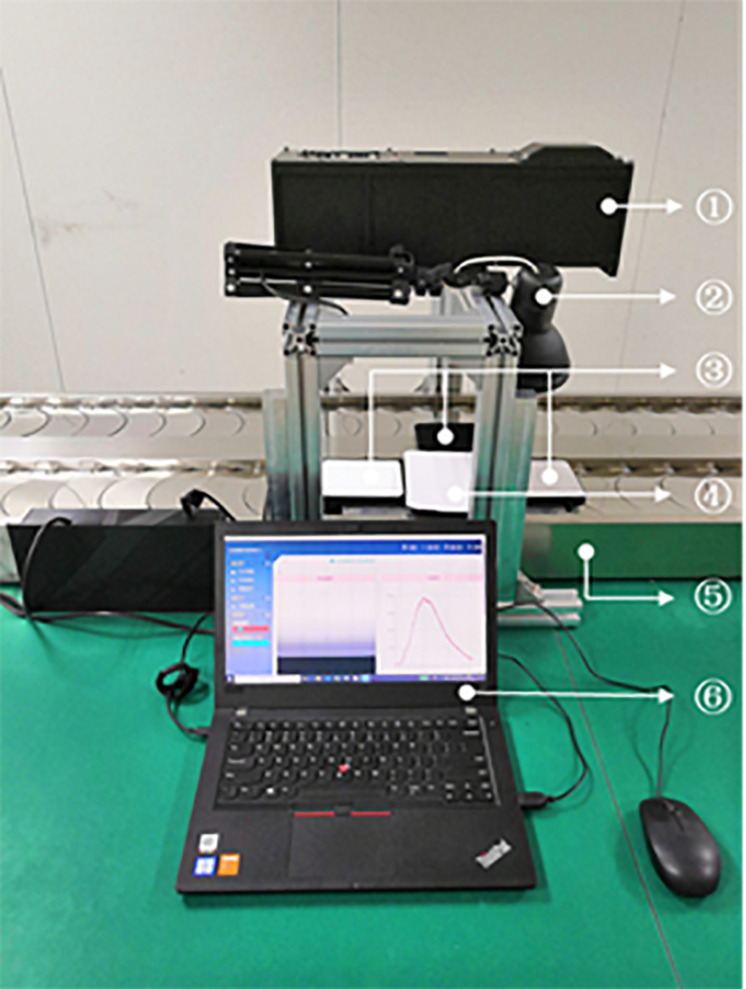
Hyperspectral image acquisition system. ① Imaging spectrometer; ② Tungsten bromide light source; ③ Standard reflectance plate; ④ Sample carrier stage; ⑤ Displacement stage; ⑥ Computer.

In this paper, hyperspectral images of wheat seeds were acquired using a combination of a pendulum sweep imaging spectrometer and a displacement platform. Pendulum sweep hyperspectral imager was developed by Chinese Academy of Sciences STS Zhejiang Center Hangzhou Center. The wavelength range of the imaging spectrometer was 400∼1000 nm, the number of spectral bands was 270, the spectral resolution was 2.6 nm, the spatial resolution was 1 mrad, and the focal length of the lens was 35 mm. As shown in Figure 1, the pendulum sweep angle of the imaging spectrometer was set to 0° in order to ensure the same light condition for each frame of data, which becomes a line-sweep type. The imaging spectrometer was placed above the displacement platform, and the line field of view was perpendicular to the running direction of the guide. The speed of the displacement stage was controlled at 0.6 m/min, the distance between the lens and the sample was 300 mm, the integration time of the detector was 16 ms, and the illumination source was a 21 V/150 W tungsten bromide light source.

When acquiring hyperspectral images, wheat seeds were placed uniformly on the sample stage above the displacement platform. A white standard reflectance plate with 98% reflectance and a black standard reflectance plate with 2% reflectance were placed at each end of the sample stage. Hyperspectral images of wheat seeds and two standard reflectance plates can be acquired simultaneously in a image as the displacement stage was moved. Combined with the imaging characteristics of the pendulum sweep imaging spectrometer, the synchronization of hyperspectral image acquisition of wheat seeds and standard reflectance plates was ensured.

**FIGURE 2:**
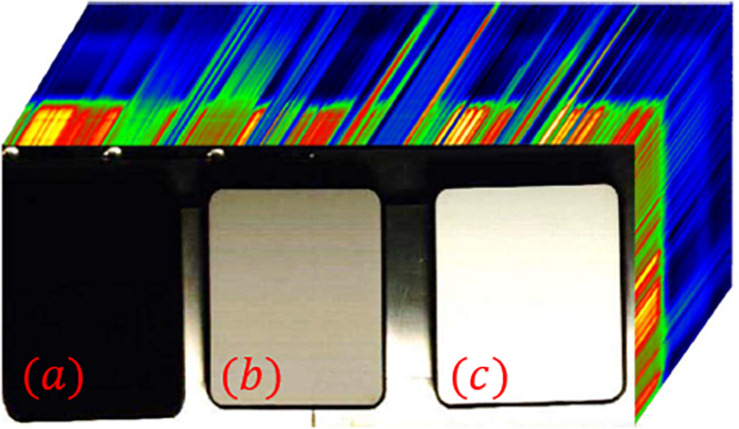
Hyperspectral images of calibrated standard reflectance plate. **(a)** reflectivity of 2% **(b)** reflectivity of 50% **(c)** reflectivity of 98%.

The standard reflectivity plates used in the experiment are shown in the Figure 2, manufactured by Labsphere. Each standard reflectivity plate has a size of 17 mm × 132 mm × 132 mm. The reflectivity of the reflectivity plates are 2, 50, and 98% from left to right.

## Methods

### Image Segmentation by Spectral Angle Combined With PCA

It can be consider that the spectral information corresponding to the pixel as a vector. The angle between the spectral information of the pixel and the x-axis of the hyperspectral image in two-dimensional plane coordinates is found according to Equation (1).

(1) $$\theta =\cos^{-1}\frac{<\vec{t},\vec{r}>}{∥\vec{t}∥\cdot ∥\vec{r}∥}$$

Where θ represents the spectral information of the pixel and the angle between the x-axis in two-dimensional plane coordinates of the hyperspectral image, which is called the spectral angle in this paper; $\vec{t}$ represents the spectral information of the pixel; and $\vec{r}$ represents the unit vector of the x-axis. The larger spectral angle indicates that the correlation between vector $\vec{t}$ and vector $\vec{r}$ is smaller. In other words, the spectral information of the pixel and the image information of the space interfere less with each other. The spectral angles of the hyperspectral images are calculated band by band, and the images and feature wavelengths corresponding to the maximum spectral angles are selected. The filtered images are added, the mean value is obtained to remove the interference of “superposition” random noise, and the image is segmented to obtain segmentation result 1 (Note: The number of feature wavelengths selected will vary for different hyperspectral images).

On the other hand, principal component analysis is applied to the corrected hyperspectral images to find the principal component images with distinct edge contours, and image segmentation is performed on it to obtain segmentation result 2. Then, the intersection of the two segmentation results is found to obtain the segmentation result 3. Finally, the corrected hyperspectral image is masked using segmentation result 3 to obtain a hyperspectral image containing only the target sample, removing the interference of excess background information.

### Hyperspectral Image Correction With Double Standard Reflectance Plates

After obtaining the hyperspectral image containing the target sample, image correction of the hyperspectral image is also required. For an image we can use a function *f*(*x*,*y*) to represent it. A hyperspectral image has one more spectral dimension than a traditional image, so a hyperspectral image can be defined by the function *f*(*x*,*y*,λ), where *x* and *y* represent two-dimensional plane space coordinates and λ represents the spectral dimension coordinates. *f*(*x*,*y*,λ) is the digital number (DN) of the pixel at any position (*x*,*y*,λ) of the hyperspectral image. It can be defined by Equation (2) as:

(2) f⁢(x,y,λ)=g⁢(x,y,λ)+ξ⁢(x,y,λ)

where *g*(*x*,*y*,λ) is the true DN of the sample point and ξ(*x*,*y*,λ) is the noise at the corresponding sample point in the hyperspectral image.

The imaging spectrometer is held stationary on top of a stand while the imaging spectrometer acquires hyperspectral images of the sample. At the same time, the sample is placed on top of a motor-controlled drive rail that moves smoothly and the field of view line slowly scans the target sample. The scanning direction of the imaging spectrometer corresponds to the x-direction in Equation (2) and the field-of-view line direction corresponds to the y-direction. Thus, when the y-coordinates are the same, the points corresponding to any x-coordinate are obtained from the same imaging region of the imaging spectrometer, which noise is extremely similar, so that the noise at these points can be considered approximately equal.

Therefore, an estimate of the noise at any sample point (*x*,*y*,λ) in the hyperspectral image can be calculated using Equation (3).

(3) $$\left\{ \begin{array}{c} g_{1}\,\left(:,y,\lambda \right)/g_{2}\,\left(:,y,\lambda \right)=R_{1}\,\left(y,\lambda \right)/R_{2}\,\left(y,\lambda \right)\\ g_{1}\,\left(:,y,\lambda \right)+\xi \,\left(y,\lambda \right)=f_{1}\,\left(:,y,\lambda \right)\\ g_{2}\,\left(:,y,\lambda \right)+\xi \,\left(y,\lambda \right)=f_{2}\,\left(:,y,\lambda \right) \end{array}\right.$$

where *R*_*1*_ and *R*_*2*_ represent the reflectance of two standard reflectance plates (known by calibration), respectively, and *f*_1_(:,*y*,λ) and *f*_2_(:,*y*,λ) are measured by the imaging spectrometer.

(4) $$\xi \,\left(y,\lambda \right)=\frac{R_{1}\,\left(y,\lambda \right)•f_{2}\,\left(:,y,\lambda \right)-R_{2}\,\left(y,\lambda \right)•f_{1}\,\left(:,y,\lambda \right)}{R_{1}\,\left(y,\lambda \right)-R_{2}\,\left(y,\lambda \right)}$$

The parameters on the right-hand side of Equation (3) are known. Therefore, the noise ξ(*y*,λ) at any sample point in different bands corresponding to the y-coordinate can be found directly. Then ξ(*y*,λ) can be subtracted from *f*(*x*,*y*,λ), so that the true DN at that sample point can be obtained for the purpose of suppressing noise in hyperspectral images.

### Method of Denoising in the Joint Spatial-Spectral Dimensional (TSG)

The Savitzky-Golay smoothing filter, developed by Savitzky and Golay, is a polynomial smoothing algorithm based on the least squares principle. First, SG filtering requires determining a window whose fixed size is (2×*m* + 1). Then the best-fit method is performed by shifting the window using least squares. Where *m* is the window coefficient of SG filtering. All the data inside the window as a collection. Let each measurement point *x* = [−*m*,1−*m*,…,0,1,…,*m*]. Finally, Equation (5) is used to fit it (Yang et al., 2017).

(5) $$p\,\left(x\right)=\sum_{k=0}^{n}a_{k}\,x^{k}$$

Where *n* is the order of the polynomial fit; *a*_*k*_ is the polynomial coefficient to be solved. The squares residuals of the fitted curve and the original spectrum are calculated, and the minimum value of the squares residuals is set as the boundary condition (Yang et al., 2017). The best coefficient matrix is found to be *B* = *X*(*X^T^*⋅*X*)^−1^*X^T^*. Then the coefficient matrix is convolved with the spectrum corresponding to each sample point in the hyperspectral image to complete the SG filtering.

The SG filtering algorithm is widely used for filtering spectral curves, and it is excellent for noise reduction and smoothing of one-dimensional curves. The one-dimensional SG convolution kernel is expanded to two dimensions, and the convolution operation is performed for each band of the image. The advantage of less information loss in SG filtering can be exploited to filter and reduce noise while fully preserving the original spatial image information. SG convolution kernel coefficients have symmetry. In order to maintain the symmetry of the convolution kernel, it is combined into a two-dimensional convolution kernel in four directions: horizontal, vertical, oblique upward and oblique downward in this paper. Then, let the size of the 2D SG convolution kernel be (2×*m* + 1)^2^ and the coordinates of each element within the convolution kernel be (x, y). The 2D SG convolution kernel can be described by Equation (6).

(6) f⁢(i,j)⁢{B⁢[|i2+j2|]/4,i⁢j⁢(i+j)⁢(i-j)=0,i,j⁢a⁢r⁢e⁢n⁢o⁢t⁢e⁢q⁢u⁢a⁢l⁢t⁢o⁢ 0⁢a⁢t⁢t⁢h⁢e⁢s⁢a⁢m⁢e⁢t⁢i⁢m⁢eB⁢[0],i=0⁢a⁢n⁢d⁢j=00,o⁢t⁢h⁢e⁢r⁢s

Where *x* = [−*m*_1_,1−*m*_1_,…,0,1,…,*m*_1_], *y* = [−*m*,1−*m*,…,0,1,…,*m*], $\left|\sqrt{i^{2}+j^{2}}\right|$ is the largest integer not larger than $\sqrt{i^{2}+j^{2}}$, and B is the one-dimensional SG convolution kernel coefficient matrix. Two-dimensional SG convolution filtering of hyperspectral images (called TSG filtering in this paper) can be accomplished by convolving the spatial images of each band in the hyperspectral image data through the two-dimensional fast Fourier transform using the two-dimensional SG convolution kernel described in Equations (6).

## Results and Discussion

### Analysis of Image Segmentation Results

**FIGURE 3:**
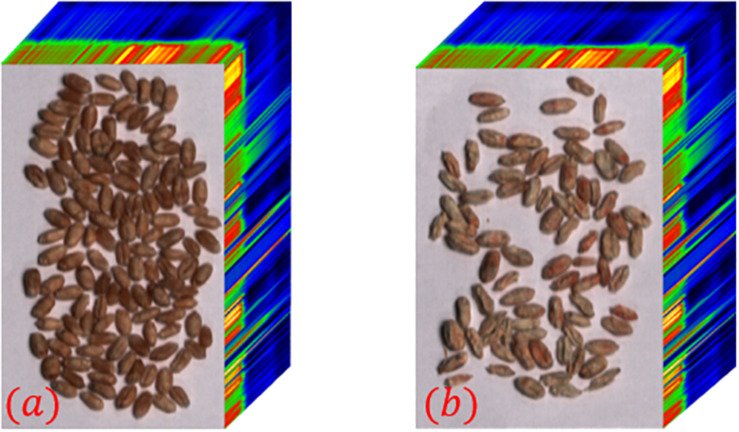
Hyperspectral images of some wheat seeds. **(a)** Sample 1–1; **(b)** Sample 1–2.

The hyperspectral images of sample 1–1 and sample 1–2 were collected as experimental data, and the hyperspectral images are shown in Figure 3. Both hyperspectral images have a wavelength range of 400∼1,000 nm, a total of 270 spectral bands, a spectral resolution of 2.6 nm, and a spatial resolution of 1 mrad. Among them, the healthy wheat seeds of Zhengmai 8 full of grains, through the body is brown. Infected with russet wheat seeds are dry, wrinkled, whitish local areas are pink or reddish brown.

**FIGURE 4:**
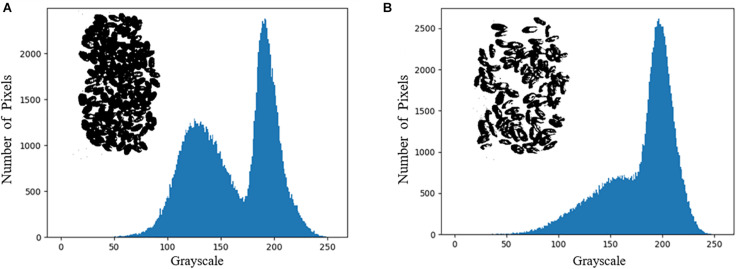
Grayscale histogram and binarized images. **(A)** Sample 1–1; **(B)** Sample 1–2.

**FIGURE 5:**
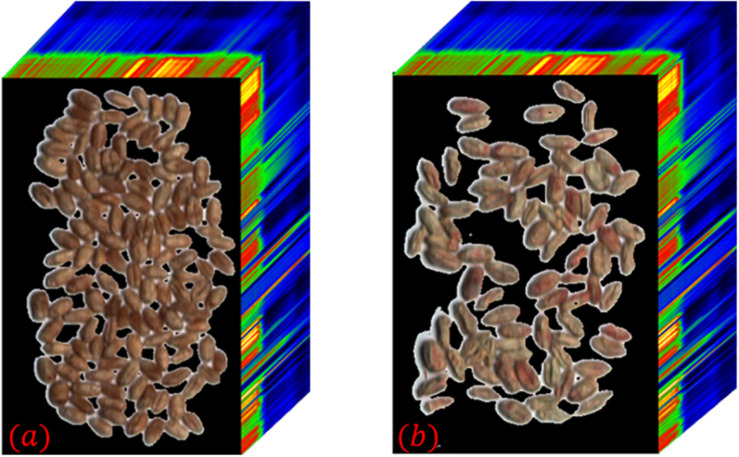
Hyperspectral images based on threshold segmentation. **(a)** Sample 1–1; **(b)** Sample 1–2.

The entropy of the hyperspectral image was calculated band-by-band, and the maximum image entropy was obtained at the wavelength of 823 nm. The image corresponding to the wavelength is 823 nm was chosen to draw a grayscale histogram and perform threshold segmentation. Based on the results of the grayscale histogram, 171 was selected as the threshold for the image at 823 nm and binarized. The grayscale histogram and the binarized image of the corresponding image are shown in Figure 4. It can be seen from Figure 4 that this method suffers from more serious interference and the deviation from the desired true threshold value exists is large. It eventually leads to the presence of many black dots in the background of the binarized image and white blocks in the wheat seed part. The segmentation of the hyperspectral image by this method and the segmentation result map is shown in Figure 5.

As shown in Figure 5, the direct application of thresholding segmentation does not achieve the desired results and there are large errors. Therefore, this paper uses the combination of spectral angle and principal component analysis to segment the hyperspectral images. After calculating the spectral angles band-by-band, the images and characteristic wavelengths corresponding to the top 20 cases of maximum spectral angles (470, 474, 479, 569, 695, 823, 878, 881, 886, 891, 894, 897, 901, 904, 907, 910, 912, 916, 918, and 922 nm) were selected in this paper. Additive operations are performed on the images corresponding to the above feature wavelengths. Image segmentation is performed on the result after finding the mean value, and segmentation result 1 is obtained.

**FIGURE 6:**
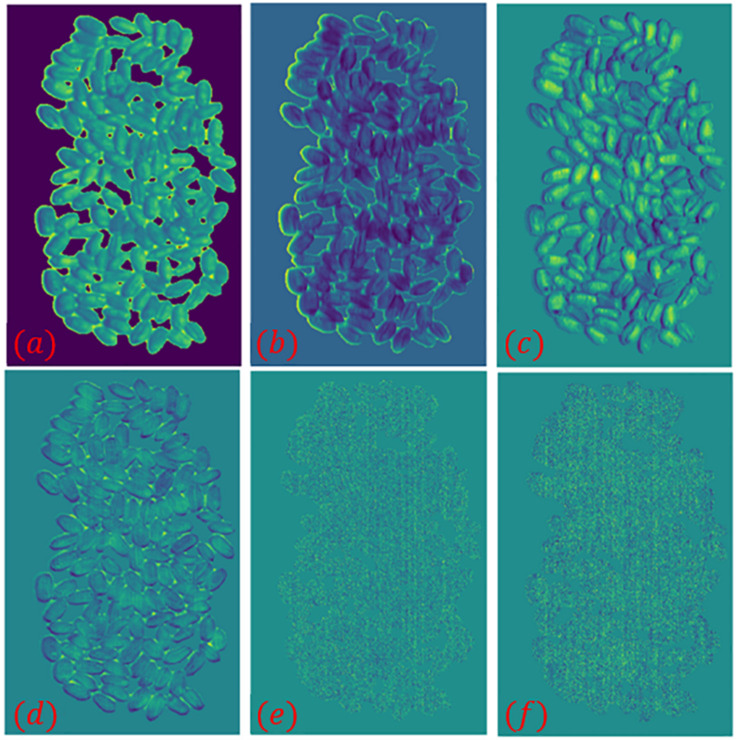
The first six principal components in the hyperspectral image of sample 1–1. **(a–f)** the pseudo-color images corresponding to the 1st–6th principal components.

On the other hand, the load distribution of the hyperspectral images of healthy wheat seeds after principal component analysis were calculated using Figure 3a as an example. The first 6 of these loadings are 95.69, 2.31, 0.68, 0.2, 0.08, and 0.04% in that order. Since the cumulative contribution of the first six principal components has reached 99.03%, the first six principal components of wheat seed hyperspectral images can be considered to represent approximately all the information of the hyperspectral images. The pseudo-color images corresponding to the first six principal components of the hyperspectral images of samples 1–1 are shown in Figure 6.

**FIGURE 7:**
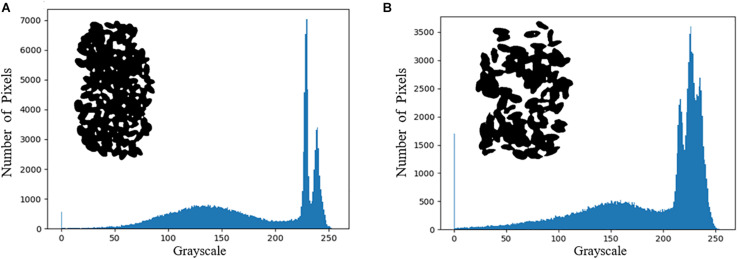
Grayscale histogram and binarized images. **(A)** Sample 1–1; **(B)** Sample 1–2.

**FIGURE 8:**
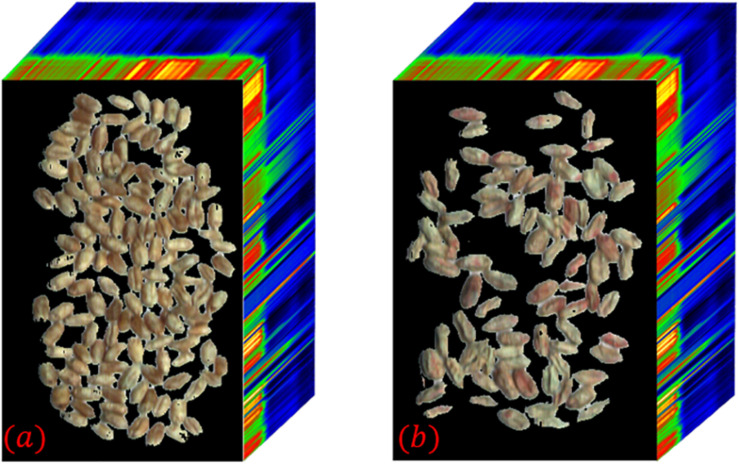
Hyperspectral images of wheat seeds after masking. **(a)** Sample 1–1; **(b)** Sample 1–2.

In the pseudo-color image shown in Figure 6, the edge part of wheat seeds in the second principal component image is highlighted, so the second principal component image is segmented and segmentation result 2 is obtained. The intersection of segmentation result 1 and segmentation result 2 is obtained as segmentation result 3. The grayscale histogram and binarized image of the splitting result 3 are shown in Figure 7. From the figure, it can be seen that the problems present in Figure 4 are solved. Finally, the segmentation result 3 is applied to mask the hyperspectral images of sample 1–1 and sample 1–2, and the final segmented hyperspectral images are shown in Figure 8.

Theoretically, the hyperspectral image of wheat seeds contains only wheat seeds with white background. However, the three-dimensional morphology of the wheat seeds and the uneven illumination of the illumination source leads to the existence of shadow parts with uneven luminance distribution around the wheat seeds, which makes the image segmentation difficult. In addition, some regions in the figure also have overlapping phenomenon, uneven distribution of light and dark in the shadow part, and insignificant abrupt change of gray value in the edge part. Moreover, the interval between adjacent wheat seeds is small, so it is not suitable for the segmentation methods commonly used in hyperspectral image segmentation.

In this paper, firstly, the images with less interference between spectral and spatial information of image elements are selected by using the spectral angle, and the interference of “superposition” random noise is removed by the addition operation. Secondly, using principal component analysis to find the principal component images of wheat seeds whose edge parts are highlighted for segmentation can reduce the misclassification rate of edge shadow parts. A more accurate segmentation result can be obtained after intersecting the two segmentation results. However, in Figure 5 we can see that there are still some background pixel points that are not segmented and the segmentation method needs further improvement.

### Results Analysis of Different Hyperspectral Image Correction Methods

**FIGURE 9:**
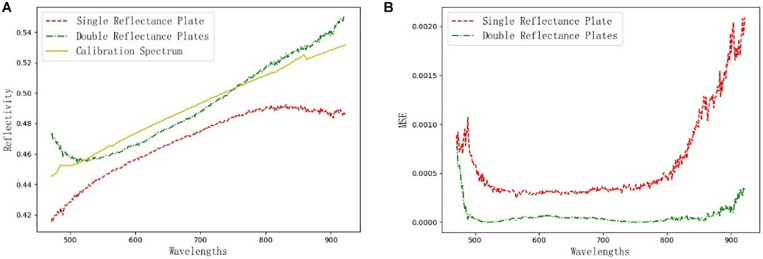
Comparison of corrected results. **(A)** Comparison of the corrected spectral curves; **(B)** Mean square error curves of different methods.

The hyperspectral image correction methods of a single standard reflectance plate and two standard reflectance plates were tested by taking the reflectance spectrum of a standard reflectance plate with 50% as the calibration spectrum. The reflectance of the one standard reflectance plate used for the black-and-white calibration experiment was 98%, and the noise of the dark current was collected by the masking lens. The reflectance of the two standard reflectance plates used in the double-plate experiment was 2 and 98%, respectively. A comparison of the results of the black-and-white correction method and the double standard reflectivity plates correction method is shown in Figure 9. The data for the calibration spectra were obtained from a standard reflectance plate with a reflectance of 50%, provided by Labsphere.

It can be seen in Figure 9A that the spectral curves obtained by calibration with the double standard reflectance plate are very close to the calibration spectra, while the spectral curves obtained by inversion with the black-and-white calibration method has large errors with the calibration spectra. To further illustrate the errors between the two methods and the calibration spectra, a comparison of the mean square error curves of the reflectance spectra and the calibration spectra obtained with different calibration methods is shown in Figure 9B. As can be seen in Figure 9B, in the wavelength range of 500–900 nm, the mean square error between the reflectance spectrum obtained by the double standard reflectance plates correction method and the calibration spectrum is less than 0.0001, which is 89.75% less than that of the black-and-white correction method. Therefore, it can be concluded that the reflectance inversion method using the double standard reflectance plate can obtain the true spectrum of the sample.

A SVM (support vector machine) (Maktabi et al., 2020; Song et al., 2020; Wang et al., 2020; Xiang et al., 2020)-based classification model for wheat blast was constructed. After retaining the first six principal components, the hyperspectral images of samples 1–1 and 1–2 were normalized and corrected by two correction methods to create the dataset. From each of them, 4,500 sample points of the hyperspectral images were randomly selected as the training set and 500 sample points as the test set. The ratio of the training set to the test set samples is 9:1. the accuracy comparison results are shown in Table 1.

**TABLE 1:** Comparison of classification accuracy of two normalized databases.

Correction	Filtering	Test set	Total	Kappa
method		classification	classification	coefficient
		accuracy	accuracy	
Black-and-white correction	No filtering	80.33%	82.63%	0.6903
Double standard reflectivity plates	No filtering	83.00%	84.51%	0.7205

It can be seen from Table 1 that the hyperspectral image classification model with bicriteria reflectance plate correction has higher classification accuracy.

Compared with the double standard reflectivity plate calibration method, the main reason for the large deviation of the black-and-white calibration method is the interference of the noise of the dark current, which fluctuates constantly under the influence of environmental factors such as temperature, and even the noise of the dark current obtained by the same instrument at different times is not consistent. Although the black-and-white correction method calculates the mean value after multiple image acquisitions, it does not guarantee the synchronization of the noise of the dark current and the hyperspectral image acquisition of the sample to be measured, and there will inevitably be errors. The double standard reflectance plate correction method acquires the hyperspectral images of two standard reflectance plates at the same time as the hyperspectral images of the sample to be measured, which ensures the continuity of the acquisition process and the consistency of the acquisition environment, so that the information closer to the real hyperspectral images can be well restored and the errors can be reduced.

### Evaluation of Hyperspectral Image Quality Before and After SG/TSG Filtering

**FIGURE 10:**
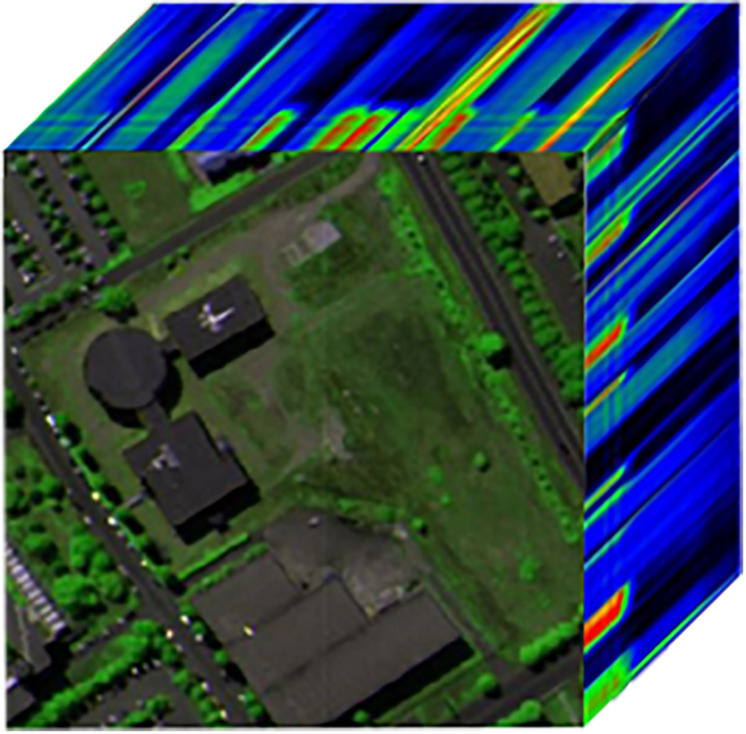
Pavia University hyperspectral image.

In this section, Pavia University hyperspectral images with a ground spatial resolution of 1.3 m and a size of 610×340 pixels are used as experimental data, containing a total of 115 bands, of which 12 bands with severe water absorption are removed (Fang et al., 2015). As shown in Figure 10, a portion of them was selected for simulation experiments, with a size of 200×200×103. The image is normalized before filtering it, and the normalization is done using the following Equation (7).

(7) ref=R(f−fmin)fref−fmin

Where *f* is the DN of the image element, *f*_*min*_ is the noise of the hyperspectral image dark current, measured by closing the lens cap, *f*_*ref*_ is the DN of the pixel corresponding to the standard reflectivity plate, *R* is the reflectivity of the standard reflectivity plate, and *ref* is the reflectivity corresponding to the pixel.

Both SG/TSG filtering algorithms have two kernel parameters of window coefficient m and order n. After optimization, the SG filtering kernel parameters *m* = 7 and *n* = 3, and the TSG filtering kernel parameters *m* = 3 and *n* = 4 are set. SG/TSG filtering was applied to the hyperspectral images of Pavia University in succession. The average image entropy, average image signal-to-noise ratio, average sharpness of each band of the hyperspectral images of Pavia University before and after comparing SG/TSG filtering, as well as the average peak signal-to-noise ratio and structural similarity of the compared original images are shown in Table 2 after retaining two decimal places.

**TABLE 2:** Hyperspectral image quality evaluation table before and after SG/TSG filtering.

Image	Average entropy	Average SNR/dB	Average clarity	Average peak SNR/dB	Structural similarity
Original image	38.76	11.11	0.21	–	–
SG filtered	39.27	15.15	0.21	55.53	99.91%
TSG filtered	39.80	15.27	0.15	34.00	96.70%

Table 2 shows that the average entropy of the hyperspectral images after SG filtering increased by 0.51, the average signal-to-noise ratio of the images improved by 4.04 dB, the average sharpness was approximately unchanged, the average peak signal-to-noise ratio was greater than 30 dB, and the structural similarity was as high as 99.91%. The average entropy of the hyperspectral images after TSG filtering increased by 1.04, the average signal-to-noise ratio improved by 4.16 dB, the average sharpness decreased by 0.06, the average peak signal-to-noise ratio was greater than 30 dB, and the structural similarity was as high as 96.70%. In summary, SG filtering preserves the image information features of hyperspectral images and enhances the image quality. TSG filtering changes the original image more than SG filtering, but still retains most of the original image features and less noise information in the filtered image. The reason for the above results is that TSG filtering not only has the ability of SG filtering to suppress the noise in the spectral dimensional of hyperspectral images, but also can filter out the noise in the spatial dimensional to achieve the effect of joint spatial-spectral dimensional filtering of hyperspectral images.

The hyperspectral images of samples 1–1 and 1–2 were processed by the image segmentation and correction method proposed in this paper. Then they are filtered by using SG/TSG filtering, and the effect of SG/TSG filtering on the accuracy of the hyperspectral classification model is investigated using the construction method and classification algorithm of the data set in 4.2. The results are shown in Table 3.

**TABLE 3:** Effect of SG/TSG filtering on the classification accuracy of hyperspectral images.

Correction	Filtering	Test set	Total	Kappa
method		accuracy	classification	coefficient
			accuracy	
Double standard reflectivity plates	No filtering	83.00%	84.51%	0.7205
Double standard reflectivity plates	SG	85.62%	86.56%	0.7459
Double standard reflectivity plates	TSG	98.48%	98.69%	0.9731

From Table 3, we can see that the TSG filtering has greatly improved the accuracy of the hyperspectral classification model, which indicates that the TSG filtering algorithm proposed in this paper can obtain good results in practical applications. The hyperspectral images of samples 1–1 and 1–2 are used as the experimental samples, and the training and test sets are constructed by the preprocessing method proposed in this paper, and the classification model is established based on the support vector machine (SVM) (Fang et al., 2015; Song et al., 2020; Wang et al., 2020; Xiang et al., 2020) algorithm, and the hyperspectral images of samples 2–1, 2–2, and 2–3 are used as the validation set.

**FIGURE 11:**
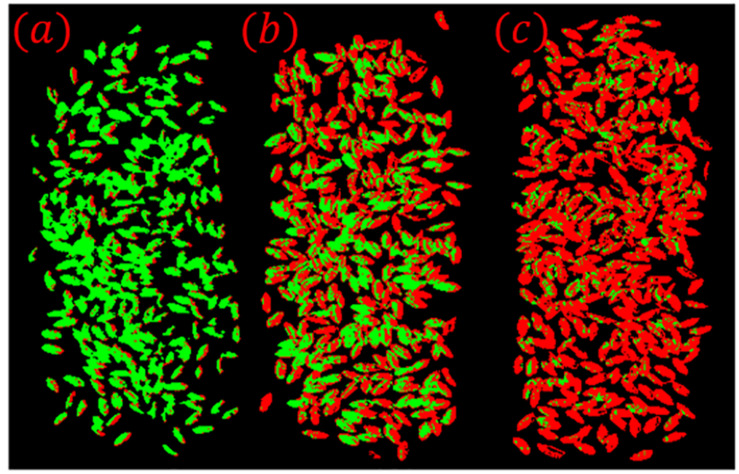
**(a)** Sample 2–1; **(b)** Sample 2–2; **(c)** Sample 2–3. Visualization of classification results for validation sets.

As shown in Figure 11, the prediction results of wheat scab identification constructed based on the hyperspectral image preprocessing algorithm proposed in this paper matched well with the actual situation and truly reflected the disease information of the samples. However, there are still a few pixels that are incorrectly identified, which can be easily identified in practical applications. On the one hand, it shows that the wheat scab classification model based on the preprocessing method which proposed in this paper has a high accuracy. On the other hand, the preprocessing algorithm needs to be improved and enhanced for the false recognition of individual pixel points.

In summary, the preprocessing method proposed in this paper has higher classification accuracy for hyperspectral images. The classification accuracy has been improved in three main ways. Firstly, the image segmentation is performed using spectral angle and principal component analysis, respectively, and the intersection is taken to obtain the segmentation result 3. The hyperspectral image is masked using the segmentation result 3 to minimize the interference of background information. Secondly, the use of double standard reflectance plates instead of black and white correction method in this paper ensures the continuity of the hyperspectral images of the samples at the time of acquisition and avoids the effect of dark current noise instability. Finally, the noise in the spectral dimensional of the hyperspectral image is suppressed while the noise in the spatial dimensional is also filtered to achieve the effect of joint spatial-spectral dimensional filtering of hyperspectral images. After the above operation, the reliability of the data set can be effectively improved and thus the recognition accuracy of the classification model can be improved.

## Conclusion

Preprocessing of hyperspectral data has a very important position in the processing and application of hyperspectral data. An effective preprocessing method can reduce or even eliminate the interference of dark currents and background of samples in the original hyperspectral data, which provides a more accurate data source for the subsequent analysis based on hyperspectral data, making the modeling accuracy more accurate and realizing the efficient and accurate utilization of data.

This paper focuses on three aspects of image segmentation, image correction and image space-spectral dimensional denoising in hyperspectral image preprocessing to make the classification accuracy of hyperspectral images improved. First, the spectral angle size is calculated band by band and the images with less interference between the spectral and spatial information of the pixels are selected. Then additive operations are performed to remove the interference of “superimposed” random noise. The principal component analysis was used to find the principal component images of wheat seeds whose edges were highlighted for segmentation, which reduced the misclassification rate of the shaded parts of the edges. After the intersection of the two results, a more accurate segmentation result can be obtained, and the interference of background information on the wheat spectral data can be effectively removed. Secondly, the standard reflectance plate with known reflectance spectra was calibrated by using double standard reflectance plates. Compared with the results of black-and-white correction, the results of the double standard reflectance plate correction are closer to the true spectrum of the sample, and the mean square error of the reflectance spectrum and the calibration spectrum is less than 0.0001. This makes the hyperspectral image acquisition continuous and improves the accuracy of the correction. Finally, TSG filtering is proposed for joint spatial-spectral dimensional denoising of hyperspectral images, which still retains most of the original image features while smoothing the spectral profile, and the filtered image has less noise information.

By comparing the total classification accuracy and kappa coefficient of the classification models with different preprocessing methods, it can be found that the preprocessing methods proposed in this paper have significantly improved the total classification accuracy and kappa coefficient. It is shown that the preprocessing method of hyperspectral images proposed in this paper can effectively improve the classification accuracy of hyperspectral images.

## Data Availability Statement

The original contributions presented in the study are included in the article/supplementary material, further inquiries can be directed to the corresponding author/s.

## Author Contributions

Y-HL: proposed the algorithm, processed the data and wrote the paper. XT: provided experimental equipment and wheat seeds and guided the revision of the paper. WZ: acquired hyperspectral images and proofread the manuscript. Q-BJ: experimental supervision. Y-XX: theoretical guidance of the algorithm. HL, Y-BZ, and LY: classification and screening of wheat seeds. Y-PF: experimental equipment setup.

## Conflict of Interest

The authors declare that the research was conducted in the absence of any commercial or financial relationships that could be construed as a potential conflict of interest.
